# A Critical Appraisal of DNA Transfer from Plants to Parasitic Cyst Nematodes

**DOI:** 10.1093/molbev/msae030

**Published:** 2024-02-15

**Authors:** Itsuhiro Ko, Olaf Prosper Kranse, Beatrice Senatori, Sebastian Eves-van den Akker

**Affiliations:** Department of Plant Sciences, The Crop Science Centre, University of Cambridge, Cambridge CB2 3EA, UK; Present address: Department of Plant Pathology, Washington State University, Pullman 99163, USA; Department of Plant Sciences, The Crop Science Centre, University of Cambridge, Cambridge CB2 3EA, UK; Department of Plant Sciences, The Crop Science Centre, University of Cambridge, Cambridge CB2 3EA, UK; Department of Plant Sciences, The Crop Science Centre, University of Cambridge, Cambridge CB2 3EA, UK

**Keywords:** horizontal gene transfer, plant-parasitic nematodes, cyst nematodes

## Abstract

Plant-parasitic nematodes are one of the most economically important pests of crops. It is widely accepted that horizontal gene transfer—the natural acquisition of foreign genes in parasitic nematodes—contributes to parasitism. However, an apparent paradox has emerged from horizontal gene transfer analyses: On the one hand, distantly related organisms with very dissimilar genetic structures (i.e. bacteria), and only transient interactions with nematodes as far as we know, dominate the list of putative donors, while on the other hand, considerably more closely related organisms (i.e. the host plant), with similar genetic structure (i.e. introns) and documented long-term associations with nematodes, are rare among the list of putative donors. Given that these nematodes ingest cytoplasm from a living plant cell for several weeks, there seems to be a conspicuous absence of plant-derived cases. Here, we used comparative genomic approaches to evaluate possible plant-derived horizontal gene transfer events in plant parasitic nematodes. Our evidence supports a cautionary message for plant-derived horizontal gene transfer cases in the sugar beet cyst nematode, *Heterodera schachtii*. We propose a 4-step model for horizontal gene transfer from plant to parasite in order to evaluate why the absence of plant-derived horizontal gene transfer cases is observed. We find that the plant genome is mobilized by the nematode during infection, but that uptake of the said “mobilome” is the first major barrier to horizontal gene transfer from host to nematode. These results provide new insight into our understanding of the prevalence/role of nucleic acid exchange in the arms race between plants and plant parasites.

## Introduction

Nematodes are the numerically dominant animals on the planet with ∼30,000 species described and perhaps more than a million postulated (Kiontke and Fitch 2013). A group of nematodes that has a strong evolutionary association with plants is the plant-parasitic nematodes (PPNs). PPNs represent a minority of nematode species but are widely studied because they constrain global food security. Cyst nematodes are some of the most devastating PPNs in agriculture. The soybean cyst nematode, *Heterodera glycines*, is estimated to cause annual losses of $1.286 billion on soybean in the United States. Cyst nematodes are sedentary endoparasites, which form prolonged biotrophic interactions with plants. Beginning with a cyst that contains hundreds of eggs, the eggs hatch after receiving the signal from roots, and a second-stage juvenile (J2) will hatch. The J2 nematode then penetrates the host root and migrates toward the vascular cylinder. Host penetration is supported by the secretion of effectors that include cell wall–degrading/modifying enzymes. Once in the vascular cylinder, these nematodes secrete other effectors, and a syncytial feeding site is formed. The feeding site forms by partial dissolution of the cell wall and fusion of the protoplast with neighboring cells into a large syncytium that can contain hundreds of nuclei. Female adult nematodes produce eggs inside their bodies, which harden and tan to form the cysts that can remain dormant in soils for decades.

It is well established that horizontal gene transfer (HGT) was a major driver of the evolution of plant parasitism in the phylum Nematoda (Danchin et al. 2010). For instance, a recent example in the potato cyst nematodes, *Globodera* spp., show that a glycosyl hydrolase family 53 gene (GH53) with high sequence similarity to GH53s from several bacterial species, instead of other nematodes, has given nematodes the ability to digest arabinogalactan (Leslie et al. 2023). As a general rule, animals do not have the capacity to digest arabinogalactan; thus, HGT is the most plausible explanation. Beyond this, several examples have been reported, which collectively demonstrate that HGT contributed to the evolution of parasitism in nematodes by providing new abilities.

Genome-wide HGT prediction algorithms, such as the most widely used “Alienness” (Rancurel et al. 2017), have been deployed extensively in PPN genome analyses (Table 1). Alienness calculates the “Alien Index (AI)” for each protein by measuring the difference in magnitude between the best nonmetazoan and the best metazoan *E*-value from Basic Local Alignment Search Tool (BLAST) results. The higher the AI, the greater the confidence that the query gene was acquired by HGT. Following identification, a phylogeny-based method is used routinely to support, and additionally filter, the BLAST-based analyses as the lowest throughput but most robust approach to identify HGT (Yang et al 2019; Siddique et al. 2022).

**Table 1:** A summary of AI results available from the published data sets of 6 PPN species, indicating the abundant putative plant-derived HGT events in those PPNs

		All possible plant-derived HGT count (AI > 0)		Highly likely plant-derived HGT count (AI > 30)	
	Source	Plant origin	Total	Plant origin	Total
Migratory ectoparasites					
*Longidorus elongatus*	Danchin et al. 2017	Not available	Not available	2	104
*Xiphenima index*	Danchin et al. 2017	Not available	Not available	3	62
Sedentary endoparasites					
*Meloidogyne incognita*	Abad et al. 2008	0	67	Not available	Not available
	Rancurel et al. 2017	89	649	1	96
	Grynberg et al. 2020	238	1,756	5	203
*G. rostochiensis*	Eves-van den Akker et al. 2016	65	519	3	91
*H. glycines*	Masonbrink et al. 2019	528	1,678	16	151
*H. schachtii*	Siddique et al. 2022	272	1,658	4	192

An apparent paradox has emerged from many such genome-wide HGT analyses. On the one hand, distantly related organisms with very dissimilar genetic structures (i.e. bacteria), and generally at present transient interactions with nematodes, dominate the list of putative donors, while on the other hand, considerably more closely related organisms (i.e. the host plant), with similar genetic structure (i.e. introns) and documented long-term associations with nematodes, are rare among the list of putative donors. This apparent paradox is at least in part explained by the difference in timescales—microbe–nematode associations likely predate plant–nematode associations by at least 100 million years.

However, parallel investigations in plant-parasitic plants have revealed large numbers of HGT events from host to parasite (Yang et al. 2016, 2019), including the incorporation of whole genes that are now passed vertically in the recipient parasite (Yang et al. 2016, 2019). In addition, multiple genes from plants have been acquired by the whitefly *Bemisia tabaci* (Gilbert and Maumus 2022). Collectively, at least in principle, plants are able to be genetic donors. Like obligate plant-parasitic plants, PPNs are dependent on the hosts to complete their entire life cycle and have intimate associations with host cytoplasm. Frequent genetic material exchange between the nematode and the host plant seems, therefore, plausible. Indeed, there are well-documented, although not fully understood, mechanisms of nucleic acid transfer between nematodes and plants, demonstrated by trans-kingdom RNA silencing (Huang et al. 2006). Although no direct evidence of DNA movement has been found between PPN and their host plants, host-derived functional HGT is plausible in PPN because of their generally similar lifestyle to parasitic plants and the shared ability of receiving foreign nucleic acid. The conspicuous absence of plant-derived HGT cases in PPNs deserves further attention.

To test the hypothesis that the transfer of nucleic acids from plants to parasitic nematodes may also be a driver of the evolution of parasitism, we critically evaluate existing plant-derived HGT predictions and expand HGT analyses to noncoding DNA, using phylogenetic and molecular biology methods. We focus on *Heterodera schachtii*, an ideal model because (i) it infects *Arabidopsis thaliana* (Sijmons et al. 1991) under sterile controlled environment, and (ii) it is a sedentary biotroph and so has a long-term association with its hosts. We discover that genome-wide HGT pipelines overpredict plant-derived HGT cases in this species. We propose a generalizable 4-step model for HGT from plant to parasite in order to evaluate why the absence of plant-derived HGT cases is observed. We find that the plant genome is mobilized by the nematode during infection, but that uptake of the said “mobilome” is the first major barrier to HGT.

## Results and Discussion

### Critical Appraisal of Plant-Derived HGT Predictions Integrated into the *H. schachtii* Genome

**Fig. 1.**
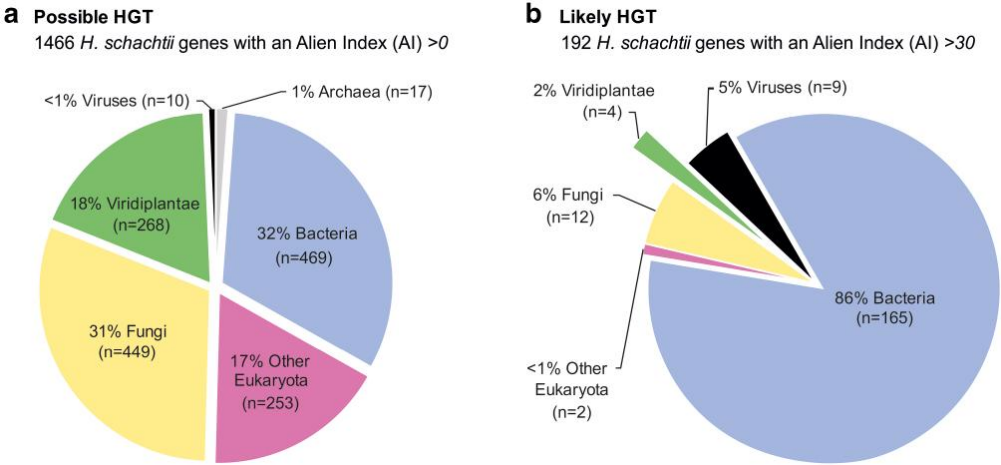
AI HGT prediction pipeline for *H. schachtii*, at 2 thresholds. a) Possible HGT events with an AI > 0. b) Likely HGT events with an AI > 30. Putative donor groups are indicated with colors: Viridiplantae (green), Viruses (black), Archaea (gray), Bacteria (blue), other Eukaryota (purple), and Fungi (yellow).

Recent genome assembly for *H. schachtii* (Siddique et al. 2022) provides an overview of predicted HGT events, typical of a cyst nematode. Of the 26,739 predicted protein-coding genes, 1,658 were identified as putatively HGT derived (Fig. 1) using the “Alienness” software (Rancurel et al. 2017). In previous studies, an AI > 30 is cited as highly likely to have an alien origin, where 30 > AI > 0 genes are recognized as possible HGT genes (Eves-van den Akker et al. 2016; Masonbrink et al. 2019; Grynberg et al. 2020). These data predict that between 2% and 18% of HGT cases may be of plant origin. Interestingly, as the AI score increased, the number of highly likely eukaryotic-origin HGT genes (including those from plants) decreased substantially (Fig. 1a and b), while the number of highly likely bacteria-origin HGT genes dominates (up to 86% from 32%). The only other category of HGT gene that increases in proportion in the highly likely HGT group is those predicted to derive from viruses.

We reassessed all putative plant-derived HGT events identified by AI, using the updated NCBI nonredundant protein sequence database (nr), in case new information since the AI calculations in Siddique et al. (2022) are informative, and combined these with phylogenetic approaches. The peptide sequence of each gene was compared to nr using BLASTp. The similar sequences returned were classified into close, distant, and putative donor groups using the following definitions: The close group included other PPN species; the distant group refers to, but not limited to, the other nonplant parasitic nematodes, fungi, bacteria, and other metazoans; and the putative donor group refers to Viridiplantae (common name “green plants”). The most similar sequences to a bona fide plant-derived HGT gene would likely reside in either the Viridiplantae and/or closely related recipient taxa that post-date the transfer event (i.e. other PPNs).

To evaluate the BLAST results, we categorized each gene with either a “pass” or “fail” for the following 3 criteria: (i) non-Viridiplantae sequences should not appear at the top BLAST results list, with the exception of closely related PPN sequences; (ii) at least 50 genes returned as BLAST results to validate a BLAST result (it was deemed unlikely that the extant descendant of the plant donor is sole descendant); and (iii) the top BLAST results must have a bitscore of >50 and identity of >30% (Pearson 2013). To prevent overlooking other high bit-score genes at the top of the blast result list, each candidate HGT gene was queried against the NCBI nr database again but excluding “viridiplantae” taxa (taxid: 33090) to revalidate criterion (i). The list of full evaluation results is available in the supplementary material S1, Supplementary Material online.

Of the 272 putative plant-derived HGT genes, 233 were unable to fulfill criterion (i) because the most similar sequence in nr was from non-Viridiplantae. The remaining 39 passed all 3 criteria but failed subsequently when criterion (i) was reevaluated by excluding “viridiplantae” during the BLAST search. The majority of those 39 candidates belonged to gene families that are ubiquitous, for example, RING/U-box superfamily protein.

According to Siddique et al. (2022), 3 cases of highly likely HGT from Viridiplantae (Fig. 1b, AI > 30) were listed as “complex,” and only Hsc_gene_18461.t1 was listed as “HGT.” However, we excluded Hsc_gene_18461.t1 as a HGT case from Viridiplantae, due to its poor HGT support from BLAST and phylogenetic analysis (supplementary fig. S1, Supplementary Material online). To understand whether the 3 criteria above were too stringent for screening HGT candidates, maximum likelihood phylogenetic trees of selected candidates were constructed based on the BLAST results.

**Fig. 2.**
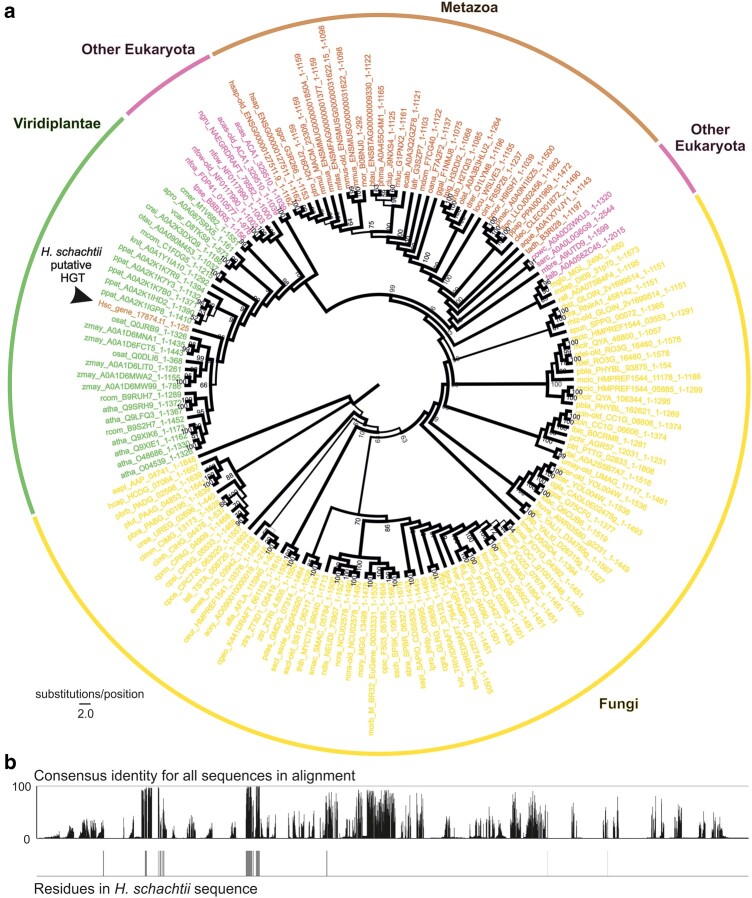
Critical appraisal of putative plant-derived HGT events. a) Cladogram inferred from the alignment of Hsc_gene_17874 (AI = 4.36) and its 150 most similar homologs from BLAST. Viridiplantae (green), Metazoa (brown), other Eukaryota (purple), and Fungi (yellow). Node labels indicate BS support values for 1,000 iterations. b) Consensus identity of the underlying alignment, with residues in the *H. schachtii* sequence indicated below.

To illustrate the approach, Hsc_gene_17874.t1 was arbitrarily selected. The phylogenetic tree inferred from 150 homologs identified by BLAST based on bit-score from the OrthoMCL-DB querying the putative HGT gene, Hsc_gene_17874.t1, is shown in Fig. 2a. Genes from Metazoa, with the sole exception being the putative HGT gene in *H. schachtii*, form a monophyletic group sister to a monophyletic group consisting of Viridiplantae and other eukaryotic genes. Genes from fungi were more distantly related. The parasite gene is deeply nested inside the Viridiplantae clade with strong bootstrap (BS) support (>94 BS support from the ancestral node shared by all Viridiplantae and this parasite gene to the node grouping the parasite gene and the rest of the plant genes together). However, this putative HGT gene resides on a long branch indicating that even though this sequence shares higher similarity with plant sequences than others, its true position is nevertheless uncertain. Moreover, manual inspection of the alignment reveals similarity across only a small proportion of the query and subject (Fig. 2b). Specifically, of the 172 residues in the tree that are >80% conserved, the *H. schachtii* sequence has a coverage of 42 (Fig. 2b). Full alignment is available in supplementary material S4, Supplementary Material online.

We additionally searched for evidence of plant-derived noncoding HGT events in *H. schachtii* and its relatives. Genomes from a range of nematode species were divided into 500 bp nonoverlapping windows and compared to a custom database of eukaryote genomes using BLASTn (species list is available in supplementary table S1, Supplementary Material online). The results provided poor evidence of potential plant-derived nonfunctional HGT cases in *H. schachtii* and other chosen PPNs: either the candidate genes belonged to a ubiquitous gene family in eukaryotes or the length of similarity was generally short (supplementary fig. S2 and table S2, Supplementary Material online). Taken together, support for plant-derived HGT is not nonexistent, but it is restricted to coding genes and not without ambiguity.

### A 4-Step Conceptual Framework for Plant-Derived HGT Events

Given the limited support for plant-derived HGT, an apparent paradox emerges: on the one hand, distantly related organisms with very dissimilar genetic structures (i.e. bacteria), and only transient interactions with nematodes as far as we know, dominate the list of putative donors, while on the other hand, considerably more closely related organisms (i.e. the host plant), with similar genetic structures (i.e. introns) and documented long-term associations with nematodes, are rare among the list of putative donors.

**Fig. 3.**
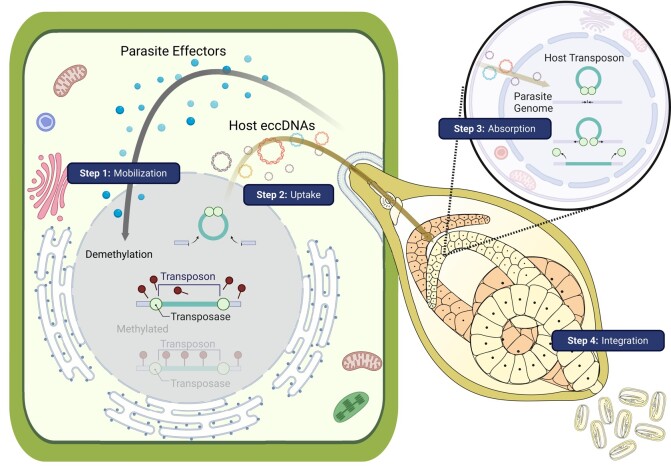
A 4-step working model for the uptake of foreign genetic material from the host plant to the parasitic nematode. Effectors (blue spheres) from the nematode (right) are delivered (black arrow) into the plant cell (left) to manipulate various aspects of biology. Ultimately, eccDNAs are taken up by the nematode (brown arrow) and integrated into the genome. Figure was created with BioRender.com.

Cyst nematode biotrophy may have existed for something in the region of 100 million years (based on 30 MYa estimates for the divergence of *Globodera pallida* and *Globodera rostochiensis* (Plantard et al. 2008; Grenier et al. 2010). In an attempt to explain the apparent absence of plant-derived HGT in cyst nematodes, a 4-step conceptual framework (Fig. 3) was established for HGT from plant hosts to parasites:

Step 1 Mobilization—Generation of mobile nucleic acids in or near the nematode induced feeding site.

Step 2 Uptake into the parasite—Mobile nucleic acids are trafficked to the parasite in some way.

Step 3 Uptake into cell/s of parasite (absorption)—Mobile nucleic acids enter the cytoplasm and ultimately the nucleus.

Step 4 Integration—Invasive nucleic acids are incorporated into the parasite nuclear genome and pass on vertically.

Considering these 4 steps as sequential hurdles, each of which may have a different probability, but all of which must presumably take place in order to result in an HGT event. This study sets out to determine whether and which criteria are met and thereby provide some explanation for the apparent conspicuous absence of plant-derived HGT events in Nematoda.

### Nematode Infection Mobilizes the Plant Genome

**Fig. 4.**
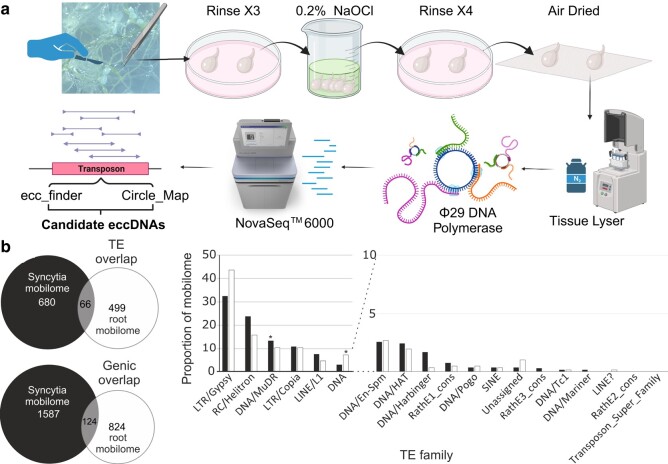
eccDNA isolation and analyses. a) Nematode tissue collection steps to avoid cross-contamination, followed by DNA extraction, RCA, sequencing, and computational analyses. Figure was created with BioRender.com. b) Left: the overlap of *A. thaliana* TEs and gene annotation on eccDNA of Col-0 syncytium (black) and root samples (white). Right: the breakdown of TE families in eccDNA of Col-0 syncytium (black) and root samples (white). Asterisks indicate those classes differentially abundant in 1 library but not in the other (Bonferroni-corrected *P* < 0.001 of hypergeometric test) compared to all TE classes in the *A. thaliana* genome: DNA/MuDR elements are depleted in control mobilomes but not syncytial mobilomes; DNA elements are depleted in syncytial mobilomes but not control mobilomes.

Regarding Step 1, mobilization, plants already have mobile DNA elements: transposable elements (TEs). More recently, it has begun to be appreciated that different types of active TEs can form extrachromosomal circular DNAs (eccDNAs; Lanciano et al. 2017). Noting that TEs, which form eccDNAs, are not the only kind of mobile element, we will nevertheless focus on them due, in large part, to previous demonstrations of mobility (Lanciano et al. 2021) and ease of detection: circular DNAs are both protected from some nucleases and can be specifically and preferentially amplified without prior knowledge on sequence using rolling circle amplification (RCA). TEs in other forms may be suitable for transfer but may require substantial advances in detection techniques to facilitate study. It is also known that cyst nematode infection results in genome-wide hypomethylation and therefore may additionally activate TEs (Hewezi et al. 2017). To test whether nematode infection does indeed mobilize the plant genome, we infected wild-type *A. thaliana* of Columbia-0 (Col-0) background (CS6000) with *H. schachtii* in vitro. Total circular DNAs were isolated from both host-infected tissues (syncytium) and adjacent developmentally matched uninfected host tissues (roots), selectively amplified using RCA, and analyzed by Illumina short-read sequencing (Fig. 4a).

Mapping the approximately 9 million pair-reads per sample identified 1,196 eccDNAs (covered length: 4,853,014 bp) in syncytium samples, compared to 955 eccDNAs (covered length: 2,227,968 bp). Comparing the composition of the mobilomes (those eccDNAs that contain TE or gene annotations), we find very little overlap between syncytia and control roots (<10% of syncytial mobilome is represented in uninfected root mobilome; Fig. 4b). We compare TE classes in mobilomes to all TE classes. Many are differentially abundant as expected (Merkulov et al. 2023). Importantly, some are differentially abundant in 1 library but not in the other (Bonferroni-corrected *P* < 0.001 of hypergeometric test) and have been highlighted in Fig. 4b: DNA/MuDR elements are depleted in control mobilomes but not syncytial mobilomes; DNA elements are depleted in syncytial mobilomes but not control mobilomes. Taken together, these data support that nematode infection does indeed differentially mobilize the plant genome. Future experiments may include a simultaneous analysis of DNA methylation and eccDNA formation to further understand the relationship between the 2. Step 1 of the 4-step conceptual framework, therefore, does not appear to be a major barrier to plant-derived HGT.

**Fig. 5.**
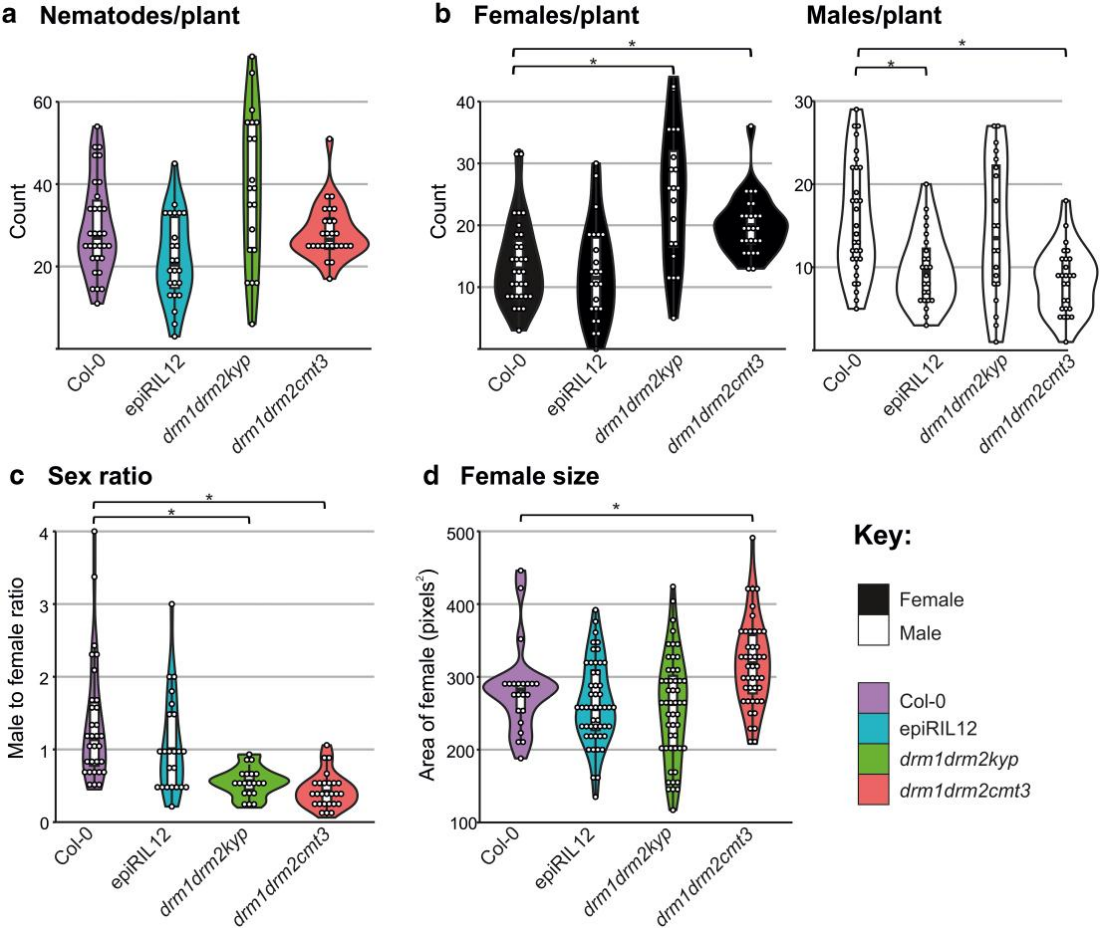
Comparing *H. schachtii* infection of Col-0 and 3 methylation mutant *A. thaliana* lines. a) Total *H. schachtii* count per plant. b) Female and male counts. c) Ratio of males to females per plant. d) Sizes of females. The difference between Col-0 and mutant lines was tested with Wilcoxon signed-rank test with Bonferroni-corrected *P*-value threshold of 0.017. Comparisons with a *P*-value below threshold are indicated with an asterisk. Each white dot represents a single infected plant (*n* = 31, 24, 20, and 26 for Col-0, epiRIL12, *drm1drm2kyp*, and *drm1drm2cmt3*, respectively).

Despite that nematode infection additionally mobilizes the plant genome, to have the best possibility of detecting the second step (i.e. uptake of genetic material into the nematode), we determined whether hypomethylated *A. thaliana* were susceptible to *H. schachtii* because they have an even greater number of mobile genetic elements. The following lines were selected: epiRIL12, the *drm1drm2cmt3* triple mutant, and the *drm1drm2kyp* triple mutant (supplementary fig. S5, Supplementary Material online). With the exception of the *drm1drm2met1-3* triple mutant, which has potentially additional pleiotropic effects (supplementary fig. S5, Supplementary Material online), the other 3 DNA methylation mutants each showed evidence of increased susceptibility as compared to Col-0. More females were observed in *drm1drm2cmt3* and *drm1drm2kyp* triple mutants than Col-0, and fewer males were found in *drm1drm2cmt3* and epiRIL12 lines (Fig. 5b). As shown in Fig. 5c, the male-to-female ratio is close to 1 in Col-0, yet significantly lower in the *drm1drm2kyp* and *drm1drm2cmt3* lines. In addition, a significant increase in female size was found in the *drm1drm2cmt3* mutant line compared to Col-0 (Fig. 5d). These data establish that these methylation mutants are indeed susceptible to nematodes, in fact generally more susceptible, specifically; more females, fewer males, and the females that do form are themselves larger.

The general increase in susceptibility of methylation mutants, noting the exception of the *drm1drm2cmt3* triple mutant, raises an interesting question on mechanism. It is not clear why/how hypomethylation would lead to increased susceptibility. It is plausible that disregulation caused by changes in methylation state contributes to a disruption of immunity-related genes (as suggested by Bennett et al. 2023), or indeed altered the expression of genes involved in feeding site formation.

### Plant Extra Chromosomal Circular DNAs Are Largely Excluded from Nematode Feeding

Having established that DNA methylation mutants are amenable to study HGT from plant to parasite, we adopted 2 approaches to address Step 2 in the working model—transfer: first, a targeted approach based on a single, well-characterized and abundant eccDNA reported in the epiRIL12 line: the EVADÉ retrotransposon (Lanciano et al. 2017), and second, a nontargeted approach sequencing all eccDNA present in infected roots and associated nematodes.

In the targeted approach, a pair of specific primers was designed for the *EVADE/COPIA93* TE, “AT5TE20395.” This TE is a good candidate because (i) it is a highly active LTR-transposon/eccDNA in the epi12RIL line (Lanciano et al. 2017), and (ii) it overlaps with a hypomethylated genomic region in *H. schachtii*-infected Col-0 *A. thaliana* (Hewezi et al. 2017), listed in supplementary table S3, Supplementary Material online. To remove cross-contamination as much as possible, we developed a bleaching method to degrade exotic nucleic acids from sample surfaces (details in the “Contamination removal” section in the Supplementary material, supplementary figs. S3 and S4, Supplementary Material online). DNA was extracted from infected roots, and females were removed from infected roots. The corresponding amplicon of eccDNA was observed in the root, leaf, and syncytium of epiRIL12 line, the leaf of *drm1*, *drm2*, *kyp* line, and Col-0. However, no amplicon of the *Arabidopsis EVADE/COPIA93* eccDNA was observed in any nematode samples either before or after the RCA (supplementary fig. S6, Supplementary Material online).

In the untargeted approach, we created mobilome-seq libraries for nematodes and infected root segments for epiRIL12 and Col-0 as described previously (supplementary table S4, Supplementary Material online). In all plant tissue mobilome-seq libraries, read coverage peaks were often located at *A. thaliana* ribosomal DNA regions, which further confirmed that the ribosomal DNA could form a circular structure as mentioned in Lanciano et al. (2017). In the nematode mobilome-seq libraries, many read pairs mapped to the *Arabidopsis* ribosomal DNA. To investigate whether those mapped reads were due to contamination during sample collection, the mapped regions were tested against the WormBase ParaSite BLAST genome data set. The BLAST results returned 99% identity sequence with the other nematode 18s ribosomal RNA sequences (e.g. *Meloidogyne hapla*, contig3097:785-2617), supporting that the mapped reads were not due to contamination or transfer but most likely endogenous parasite cross mapping.

**Fig. 6.**
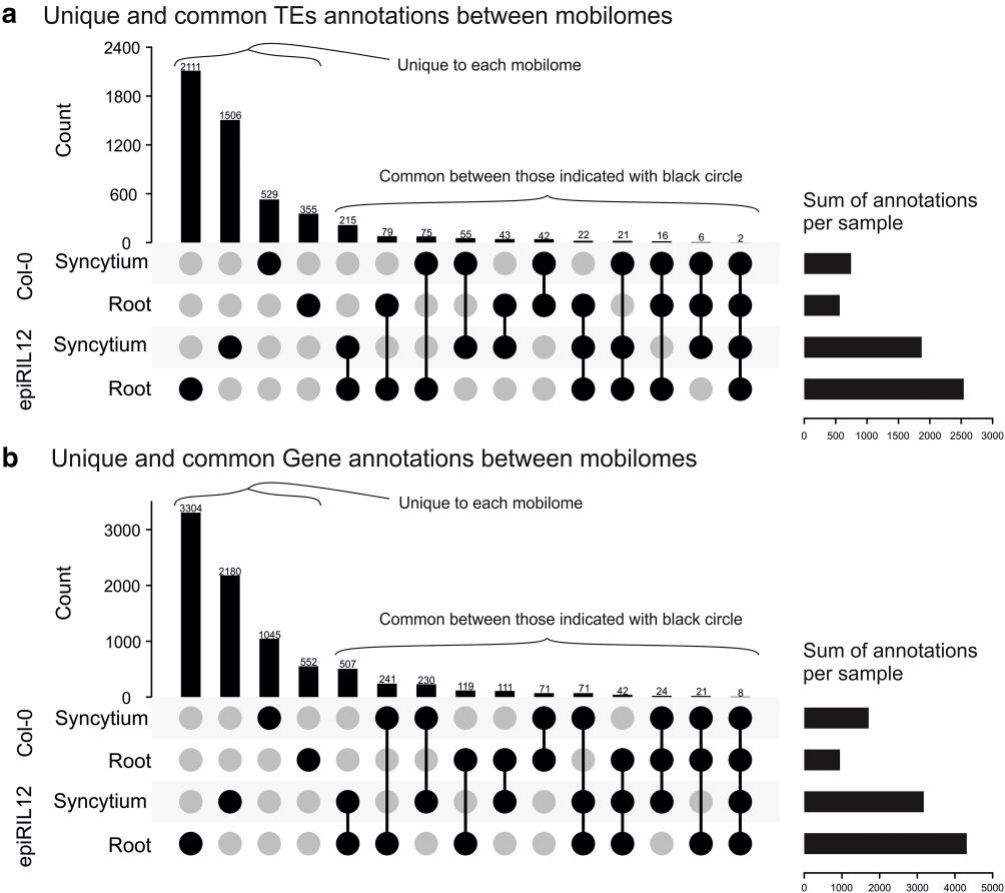
A comparison of unique and common annotations in eccDNAs. Upset plots are shown for a) TE annotations within eccDNAs or b) gene annotations within eccDNA libraries of syncytium and uninfected root for Col-0 and epiRIL12 if they do not overlap with TE annotations. Black circles indicate which samples are presented in the bar above. Black circles joined by a line indicate those annotations share between samples. The chart on the right shows the total number of annotations for each library.

In the *Arabidopsis* mobilome-seq libraries, divergent eccDNA profiles were found among the epiRIL12 and Col-0 line samples. The global eccDNA landscape of epiRIL12 is drastically different to Col-0: it has many more eccDNAs than Col-0 overall; infected and uninfected epiRIL12 roots are more similar to one another than infected epiRIL12 is to infected Col-0; and epiRIL12 has more eccDNAs in uninfected roots than in infected roots (Fig. 6). epiRIL12 uninfected root shares more eccDNAs with Col-0 syncytia than it does with Col-0 uninfected root (in both TEs and genes). By comparing the previously identified *A. thaliana* eccDNA data sets in Lanciano et al. (2017) and Wang et al. (2021) to our *A. thaliana* mobilome-seq libraries, we found roughly 10% overlap. Taken together with the almost complete absence of overlap between the 4 plant samples sequenced herein, it illustrates the extremely dynamic nature of eccDNA production under different conditions.

Among all the nematode mobilome-seq libraries, female *H. schachtii* infected in the epiRIL12 line was found with the most abundant eccDNAs both in TEs and coding gene regions (supplementary fig. S7, Supplementary Material online). Similar to the plant mobilome-seq libraries, little overlap was found between samples.

**Fig. 7.**
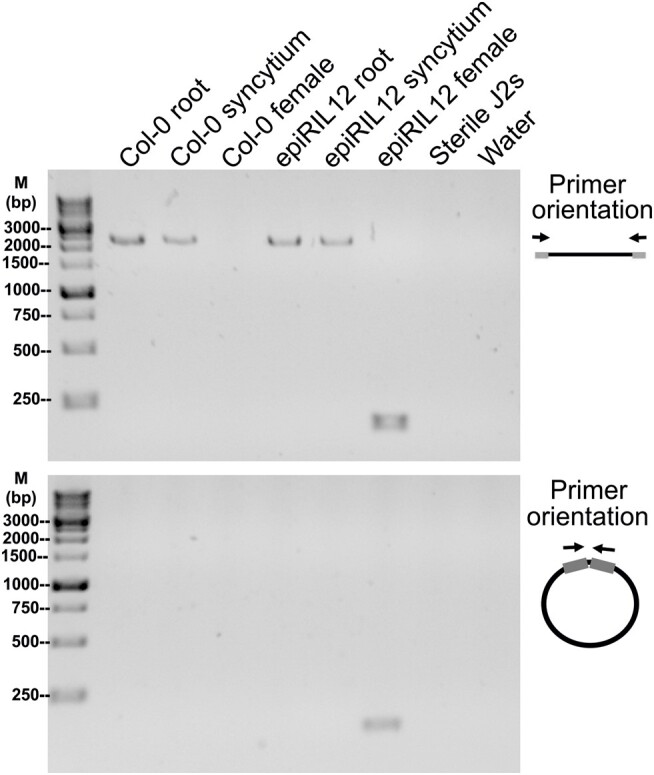
PCR amplification of *A. thaliana* COPIA61 eccDNA from plant and nematode samples. The top shows amplification of the linear element, while the bottom shows amplification of the circular element. M represents the marker.

In total, 215 reads from eccDNA libraries of female nematode parasites on epiRIL12 were mapped to AT2TE07175 (name: AT2G04490) corresponding to an eccDNA identified by circle_map alone (chromosome 2, start: 1,559,133; end: 1,561,452). AT2TE07175 belongs to COPIA61 LTR-retrotransposon family, compositing 4,986 bp long. No reads from the other nematode libraries were mapped to the same region except the epiRIL12 plant tissues, which suggests this unique sequence only presented in the female nematode parasitized on epiRIL12 line could be a case of eccDNA transfer. To confirm the presence of this *A. thaliana* eccDNA in the nematode, inbound and outbound polymerase chain reaction (PCR) primers were designed to amplify the linear and circular forms of *A. thaliana* COPIA61 eccDNA. A DNA fragment around 150 bp was amplified by both primers in the epiRIL12 female nematode sample that were used for mobilome-seq (Fig. 7), whereas only linear form of COPIA61 was amplified in epiRIL12 root and syncytium samples with the amplicons' size around 2,500 bp. The Sanger sequencing result of the amplicon from epiRIL12 female nematode sample aligned to roughly 100 bp of the AT2TE07175 sequence. BLAST search of this amplicon sequence was performed against NCBI nr nucleotide data set and the WormBase ParaSite BLAST genome data set. As a result, multiple *A. thaliana* chromosome 2 genome regions were returned as the best homologs with 100% identity and 100% coverage to the query sequence, whereas no homolog was found in the nematode data set. In summary, the fragment presented in both mobilome-seq and PCR results in epiRIL12 female nematode sample was most likely derived from *A. thaliana* COPIA61 regions, after we stringently evaluated the possibility of a contamination or an artifact (e.g. primer dimer). However, we were not able to explain why only partial COPIA61 regions were presented in the female nematode sample, and this mapped eccDNA was not found in either the Lanciano et al. (2017) or Wang et al. (2021) database of identified eccDNA in *A. thaliana*.

The fact that uptake appears to be the first major barrier may have implications outside the immediate goals of this research. Future research to understand the nature of the barrier may inform our understanding of the nature of the parasite in general and indeed routes to control. It is well established that nematodes can uptake some mobile ribonucleic acids from the plant host, but the rules governing this are not established. It is plausible that a greater understanding of the lack of uptake of deoxyribonucleic acids will inform the boundary conditions of the so-called host-induced gene silencing.

## Conclusion

In this study, we adopted the phylogenomic method for in-depth plant-derived HGT validation in PPNs and the real-time molecular biology approaches to uncover the potential pathway of nucleic acid transfer. These analyses provide limited support that nematodes obtain genes from plants and limited support of the real-time transfer of eccDNA between the plant and nematode—much less than anticipated based on previous reports and the “you are what you eat” model described in Yang et al. (2016) and Haegeman et al. (2011). It is possible that the number of putative plant-derived HGT events will grow with an increased coverage of genome sequences in the donor and recipient phyla—but it is not anticipated that this source of HGT would outnumber those derived from bacteria.

We aimed to provide an explanation for the apparent conspicuous absence of plant-derived HGT in parasitic nematodes by proposing and testing a 4-step model for HGT. We demonstrated that the first step of the model is not a barrier: plant-parasitic nematodes literally mobilize the host plant genome during infection. The second step is clearly the first major barrier (although we provide some evidence that it may be surmountable). Together with subsequent barriers of unknown probability that, whatever their likelihood, would presumably be multiplicative rather than additive, these data may also help to explain the apparent absence of plant-derived HGT in cyst nematodes.

## Materials and Methods

### BLAST and Phylogenetic Analysis

Unless otherwise stated, BLAST analyses were performed from June 2020 to December 2021 as described. BLASTp and BLASTn (Boratyn et al. 2013) searches were carried out at 3 different online servers with their respective databases: (i) the NCBI nonredundant protein and nucleotide databases at https://blast.ncbi.nlm.nih.gov/Blast.cgi using the relevant parameters; (ii) 150 core species proteomes database at OrthoMCL (https://orthomcl.org/orthomcl/app/search/sequence/ByBlast) with *E*-value of 1e−05 and ≥50% of percent match length (Li et al. 2003); and (iii) the protein database at WormBase ParaSite (https://parasite.wormbase.org/Multi/Tools/Blast), using the default parameters (Howe et al. 2017). Any specific parameters are reported for each experiment. The first 100 BLAST results, in the order in which BLAST presents them, were collected. MUSCLE (v3.8.31) was used to generate protein alignment with default parameters (Edgar 2004). Model prediction and un/mid-point-rooted phylogenetic trees were inferred from each alignment by using IQ-TREE (with default parameters and 1,000 ultrafast BSs; Trifinopoulos et al. 2016).

#### Bit-Score–Based Screening (500/1,000 bp Windows)

Genome and annotation data were retrieved from the NCBI, WormBase ParaSite, Phytozome, and Ensembl databases from August 2020 to June 2021. A python script was written to divide query genomes into 500 bp nonoverlapping windows. Each window was then tested against the local customized database (supplementary table S1, Supplementary Material online) using BLASTn (version 2.10.1+) with an *E*-value setting of 1e−1. An R script was designed to compare the bit-score of donor group and distant group for each genome window, and a scatter plot was made to visualize the entire query genome results. The HGT candidate windows are identified by the following criteria: (i) the best bit-score in donor lineage is at least 50 higher than the best bit-score in distant lineage, and (ii) the alignment is at least 100 bp in length for both query and subject.

### Plant Growth and Nematode Inoculation

*Arabidopsis thaliana* ecotype Col-0 (CS60000) and 3 triple methylation mutant seeds (*drm1, drm2, cmt3*, CS16384; *drm1, drm2, met1-3*, CS16387; and *drm1, drm2, kyp*, CS16388) were obtained from the Nottingham Arabidopsis Stock Centre (NASC). epiRIL12 line seeds were provided by Dr. Marie Mirouze, IRD, France.

The *Arabidopsis* seeds were sterilized by 1:5 dilution of household bleach (Parazone) solution (1% [v/v] NaOCl solution) for 20 min with gentle shaking. Seeds were rinsed with Ultrapure ddH_2_O 7 times in the flow food. The sterilized seeds were grown on 5 cm KNOP media plates. Plates with seeds were stored at 4 °C in the dark for 2 d to ensure uniform germination. *Arabidopsis* plates were grown in a growth cabinet at 21 °C under a long daylight condition with 16 h of light and 8 h of darkness. Approximately 100 J2 *H. schachtii* were dispensed evenly on the surface of the media above the root area for each plate at approximately 14 d post germination. After inoculation, the plates were returned to the growth cabinet.

### Measurement of Nematode Infection and Statistical Analyses

Female and male nematodes on infected *Arabidopsis* plates were counted at approximately 14 d post infection on a dissecting GX Microscope (GT Vision). A Raspberry Pi High Quality Camera with 3D printed “imaging tower” was used to capture the root infections (Kranse et al. 2022), and ImageJ was used to measure the size of females that feed on different *Arabidopsis* lines. To test the difference between nematode infection on mutant and Col-0 *Arabidopsis* lines, a Wilcoxon rank-sum test with continuity correction was performed on R. A Bonferroni-corrected *P* = 0.017 was used for the cutoff for Wilcoxon test to account for multiple (3) comparisons.

### Tissue Collection

After 22 to 30 d post infection, female cyst nematodes and *Arabidopsis* tissues were collected from sterile tissue culture. For the PCR experiment, equivalent tissues were collected from Col-0 and eipRIL12, which included uninfected root segments, females, infected root segments (approximately 0.5 cm upstream and downstream of syncytium), leaves, and flowers. For the eccDNA-seq library construction, the following 7 tissue types were collected: segments of the root containing the syncytium (in each of epiRIL12 and Col-0); adjacent uninfected and developmentally matched segments of roots (in each of epiRIL12 and Col-0); female *H. schachtii* feeding on the collected syncytium tissues (from each of epiRIL12 and Col-0); and sterile *H. schachtii* juveniles (as a negative control). Samples were collected as in supplementary fig. S8, Supplementary Material online. Each tissue collection had 2 biological replicates. Nematode and syncytium tissues were immersed in 0.2% NaOCl solution for 5 min to remove possible external contamination (detailed in the “Contamination removal” section in the Supplementary material; supplementary figs. S3 and S4, Supplementary Material online). Treated tissues were rinsed with sterilized water 7 times, stored in 2-mL Eppendorf Safe-Lock tubes, and frozen in liquid nitrogen.

### Total DNA Extraction from Plant and Nematode Tissues

Frozen tissues were disrupted by TissueLyser (QIAGEN). One 5 mm and one 2 mm glass beads were added to the tubes to facilitate the grinding process. The disruption and homogenization were carried out at an oscillation frequency of 30 Hz for 2 min. Disruption was repeated until the tissue became a fine white powder. The samples were recooled in liquid nitrogen after each round of grinding.

The resultant power of plant tissue was resuspended in 800 µL of CTAB (SERVA) with 4% (w/v) PVP-40 and 75 µL Proteinase K (20 mg/mL). After vortexing vigorously, the resuspended solution was incubated in an Eppendorf ThermoMixer at 65 °C with 700 rpm shaking for 1 h. To remove the RNA, 15 µL RNase A was added to the lysed solution with 5 min of incubation at room temperature.

The resultant powder of nematode tissue was resuspended in 400 µL lysis buffer (0.1 M Tris [pH 8.0], 0.5 M NaCl, 50 mM EDTA, and 1% SDS) and 100 µL Proteinase K, vortexed, and incubated at 55 °C with 700 rpm shaking for 24 h. Finally, 25 µL of RNase A was added to the lysed solution with 5 min of incubation at room temperature.

Four hundred microliters of phenol:chloroform:isoamyl alcohol (25:24:1, PCI) was added to each sample (plant or nematode), vortexed vigorously for 2 min, and centrifuged at approximately 13,000 × *g* for 10 min. The top aqueous solution was carefully moved to another tube without the PCI phase, and 400 µL of PCI was added to the remaining solution to repeat the previous step. The final aqueous solution was mixed with 800 µL of chloroform:isoamyl alcohol (24:1), and the vortex and centrifugation steps were repeated.

The top aqueous solution was moved to a new tube and mixed with 0.1 volume of 3 M NaOAc solution, 2 µL of UltraPure Glycogen (Invitrogen), and 1 volume of 100% EtOH. The extracted DNA solution was then incubated at −20 °C for 24 h and centrifuged at 12,000 × *g* for 60 min at 4 °C. After removal of all the supernatant, the pellet was resuspended in 500 µL ice-cold 70% EtOH and centrifuged at 12,000 × *g* for 30 min. The clean pellet was collected, air dried at room temperature, and dissolved in 30 µL of Ambion Nuclease-Free Water (Invitrogen).

### PCR

PCR was performed in 50 µL reaction volumes following the manufacturer's instructions. Each reaction included 1 unit of Q5 High-Fidelity DNA Polymerase (NEB), 0.5 µM of each forward and reverse primer, 200 µM of dNTPs, and 10 µL of 5X Q5 reaction buffer. One microliter of template was added to each reaction (at least 1 ng of DNA input except the no template control). Ambion Nuclease-Free Water (Invitrogen) was used to adjust the final volume and as the no template control. The primers used in this study are listed in supplementary table S5, Supplementary Material online.

PCR cycling conditions were as follows: 98 °C for 3 min, followed by 30 cycles of initial denaturing phase at 98 °C for 10 s, annealing at relevant temperature (supplementary table S5, Supplementary Material online) for 30 s, and an extension phase at 72 °C for 2 min. A final extension phase was operated at 72 °C for 2 min.

For gel electrophoresis, the PCR product was mixed with 5X GelPilot DNA Loading Dye (QIAGEN) to make a final concentration of 1X and loaded to a 1.5% (w/v) agarose gel, including 1X Tris–Borate–EDTA (TBE) solution and 1% (v/v) Midori Green Advance DNA stain (NIPPON Genetics). The gel was electrophoresed at 100 V for sufficient time until the bands separated. G:BOX (SYNGENE) was used to image the gel.

When verification of the amplicon was needed, the amplicon of interest was excised from the gel, and the DNA was purified using the Monarch DNA Gel Extraction Kit (NEB). The concentration of the purified DNA was measured by Qubit dsDNA BR Assay Kit (Thermo Fisher Scientific) before sending for Sanger Sequencing service provided by GENEWIZ, United Kingdom.

### eccDNA Enrichment and Total DNA Sequencing

The 25 µL total DNA isolated from plant or nematode tissues was treated with PlasmidSafe DNase (Epicentre) following the manufacturer's instructions, except the reaction was incubated at 37 °C for 17 h. Digested DNA samples were cleaned and precipitated following ethanol as described previously, except the DNA pellet was resuspended in 5 µL of TempliPhi sample buffer instead of water. eccDNA as well as other circular DNAs was amplified from the resuspended DNA solution through RCA by using the Illustra TempliPhi Kit (GE Healthcare). The reaction was performed according to the manufacturer's instructions, and the incubation for RCA was performed at 28 °C for 72 h to maximize the amplification. The Phi29 enzyme used for RCA reaction was deactivated by incubating the sample at 70 °C for 30 min. For both *A. thaliana* and *H. schachtii* samples (listed in supplementary table S3, Supplementary Material online), 400 ng of input DNA was sent to Novogene, Cambridge, United Kingdom, for 150 bp paired-end TruSeq Library preparation and sequencing. DNA was performed on a NovaSeq 6000 (Illumina).

### Data Analysis, Read Mapping, and eccDNA Identification

Data analysis followed the procedure described in Lanciano et al. (2017) with the following modifications. The Illumina NGS sequence reads (FASTQ files) were first checked with FastQC tool (v 0.11.9; Andrews 2018), followed by Trimmomatic (v.0.39; Bolger et al. 2014) to filter the adaptors and low-quality bases: TruSeq3-PE-2.fa:2:30:10. To remove any reads from *Arabidopsis* plastid DNA, reads were mapped to the *A. thaliana* mitochondrial genome (Y08501.2) and chloroplast DNA (AP000423.1) using Bowtie2 (v2.3.5.1; Langmead and Salzberg 2012) with sensitive local mapping. Any unmapped reads were then mapped to the combined reference genome (*A. thaliana* TAIR10 genome from The Arabidopsis Information Resource, http://www.arabidopsis.org, and *H. schachtii* genome, Cam_v1.2; Siddique et al. 2022). The parameters used for this mapping process were sensitive local mapping; no allowance for multiple mapping (-k 1); and output file as sam. The output sam file for each sample was sorted, indexed, and converted into a bam file using samtools (v1.7; Li et al. 2009). bamCoverage (v3.5.0; Ramírez et al. 2016) was used to generate a read coverage track (BigWig file) for each bam file, and the sequence read enrichment on the reference genome for each sample was visualized with the Integrative Genomics Viewer (IGV) software (v. 2.10.3; Robinson et al. 2011).

eccDNAs were identified by using the default settings of Circle_Map (v1.1.4; Prada-Luengo et al. 2019) and eccFinder (Zhang et al. 2021). To obtain reliable results from Circle_Map, we only kept the eccDNAs that have at least 2 split reads and a circle score that is above 10. eccFinder was used to identify the region of the genome with both split and discordant reads, indicative of circular DNA. To evaluate eccDNA profiles accurately, only the eccDNA regions identified in both tools were used for further analysis. A full list of overlap regions is available in the supplementary materials S2 and S3, Supplementary Material online. The sam alignment file from each sequence library was used as an input. The output file was a bed file that only contained the information of identified circular DNA for each sample. Bedtools (v2.30.0; Quinlan and Hall 2010) intersect module was used to identify any eccDNAs, which overlapped with the *A. thaliana* TE database based on TAIR10, established by Hadi Quesneville (www.arabidopsis.org). TBtools was used to generate upset charts and Venn diagrams for this study (Chen et al. 2020).

## Supplementary Material

Supplementary material is available at *Molecular Biology and Evolution* online.

## Supplementary Material

msae030_Supplementary_Data

## Data Availability

The mobilome-seq libraries generated in this study have been deposited at the NCBI database (https://www.ncbi.nlm.nih.gov/bioproject/) under BioProject ID PRJNA1020889.
