# Supplementary material for: A Critical Appraisal of DNA Transfer from Plants to Parasitic Cyst Nematodes
Source: Mol Biol Evol. 2024 Feb 15;41(2):msae030. doi: 10.1093/molbev/msae030 (PMC10899095; doi:10.1093/molbev/msae030)
Supplement: msae030_Supplementary_Data [file msae030_supplementary_data.zip › ko_Supplemental_files_final_SEVDA_revised.pdf]

Supplemental files

A critical appraisal of DNA transfer from plants to parasitic cyst nematodes

Itsuhiro Ko[1,2], Olaf Prosper Kranse[1], Beatrice Senatori[1], Sebastian Eves-van den Akker[1]

[1] The Crop Science Centre, Department of Plant Sciences, University of Cambridge, Cambridge CB2 3EA, UK

[2] Department of Plant Pathology, Washington State University, Pullman, 99164, USA.

Taxa	RGB	Colour
Viridiplantae	0 204 0	Green
Metazoa	0 0 205	Blue
Fungi	255 153 0	Orange
Other Eukaryota	0 0 0	Black

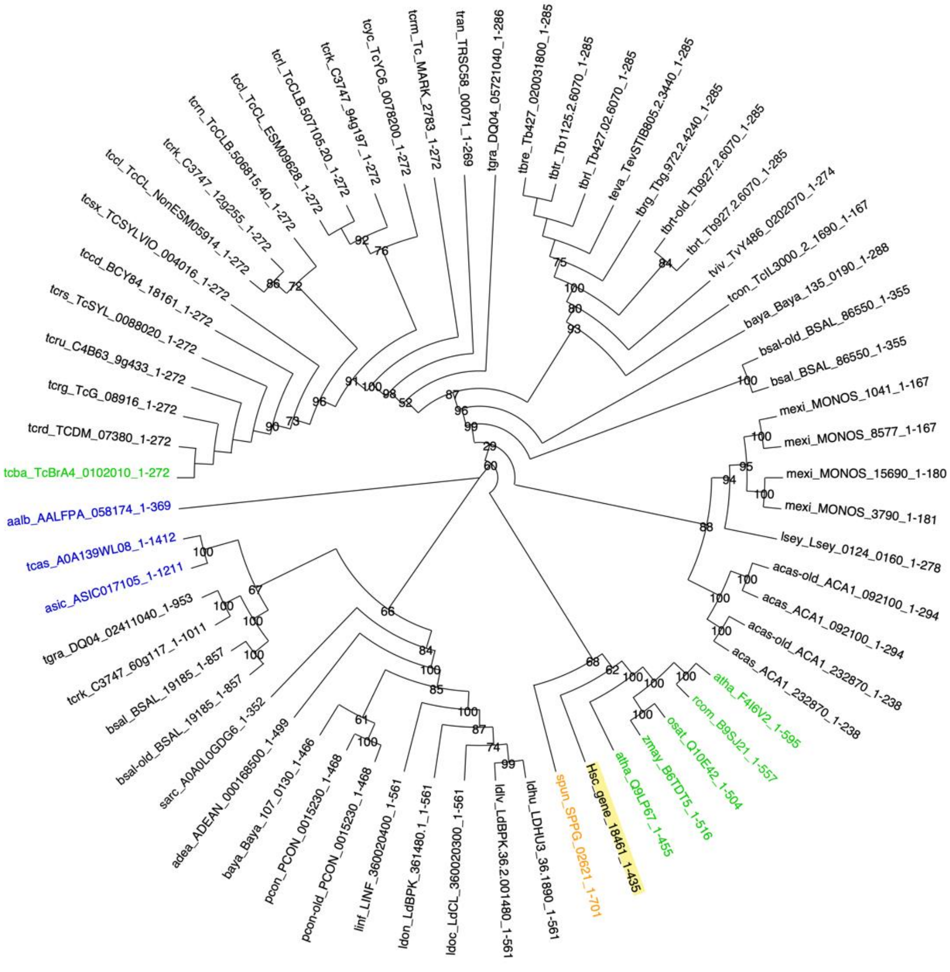

Figure S1. A phylogenetic tree of Hsc_gene_18461.t1, a putative HGT gene with the highest AI score (AI=44.85). A BLAST search against the OrthoMCL DB querying Hsc_gene_18461 only yielded 61 results. The parasite gene grouped outside a monophyletic clade of viridiplantae genes with moderate bootstrap support (62), and a fungal gene is the next closest sister to them. The phylogeny suggests that instead of a horizontally acquired gene, Hsc_gene_18461 likely belongs to a ubiquitous gene family present in many organisms.

Query genome	Wormbase ParaSite		Donor lineage (Plants)	Pytozome V13		Distant (Non-plant eukaryotes)	Wormbase ParaSite + NCBI
Heterodera schachtii	PRJNA722882	Rosid	*Arabidopsis thaliana*	TAIR 10	Nematode Clade IV	*Bursaphelenchus xylophilus*	PRJEA64437.WBPS15
Meloidogyne incognita	PRJEB8714.WBPS15	Asterid	*Solanum lycopersicum iTAG2.4*	514.SL3.0	Animal	*Mus musculus*	GCF_000001635.27_GRCm39
Globodera rostochiensis	PRJEB13504.WBPS15	Rosid	*Fragaria vesca*	501_v2.0	Nematode Clade IV	*Strongyloides stercoralis*	PRJEB528.WBPS15
Heterodera glycines	PRJNA381081	Basal	*Beta vulgaris*	548_EL10_1.0	Insect	*Drosophila melanogaster*	GCF_000001215.4
Meloidogyne arenaria	PRJNA438575	rosid	*Vitis vinifera*	457_Genoscope.12	Eukaryote	*Saccharomyces cerevisiae S288C*	GCF_000146045.2_R64
Meloidogyne arenaria	PRJEB8714	Monocot	*Sorghum bicolor*	454_v3.0.1.fa	Nematode Clade I	*Romanomermis culicivorax*	PRJEB1358
Caenorhabditis elegans (Pusedo control)	PRJNA13758.WBPS15	BA	*Amborella trichopoda*	v1.0	Nematode Clade V	*Pristionchus pacificus*	PRJNA12644
		Monocot	*Musa acuminata*	v1	Animal	*Strongylocentrotus purpuratus*	GCA_000002235.4 Spur_5.0

Table S1. List of genome resources. These plant species were chosen because they are representative species in flowering plants with relatively complete genome data, which were previously used for research of HGT events between plant-parasitic plants and their hosts (Yang et al. 2016)

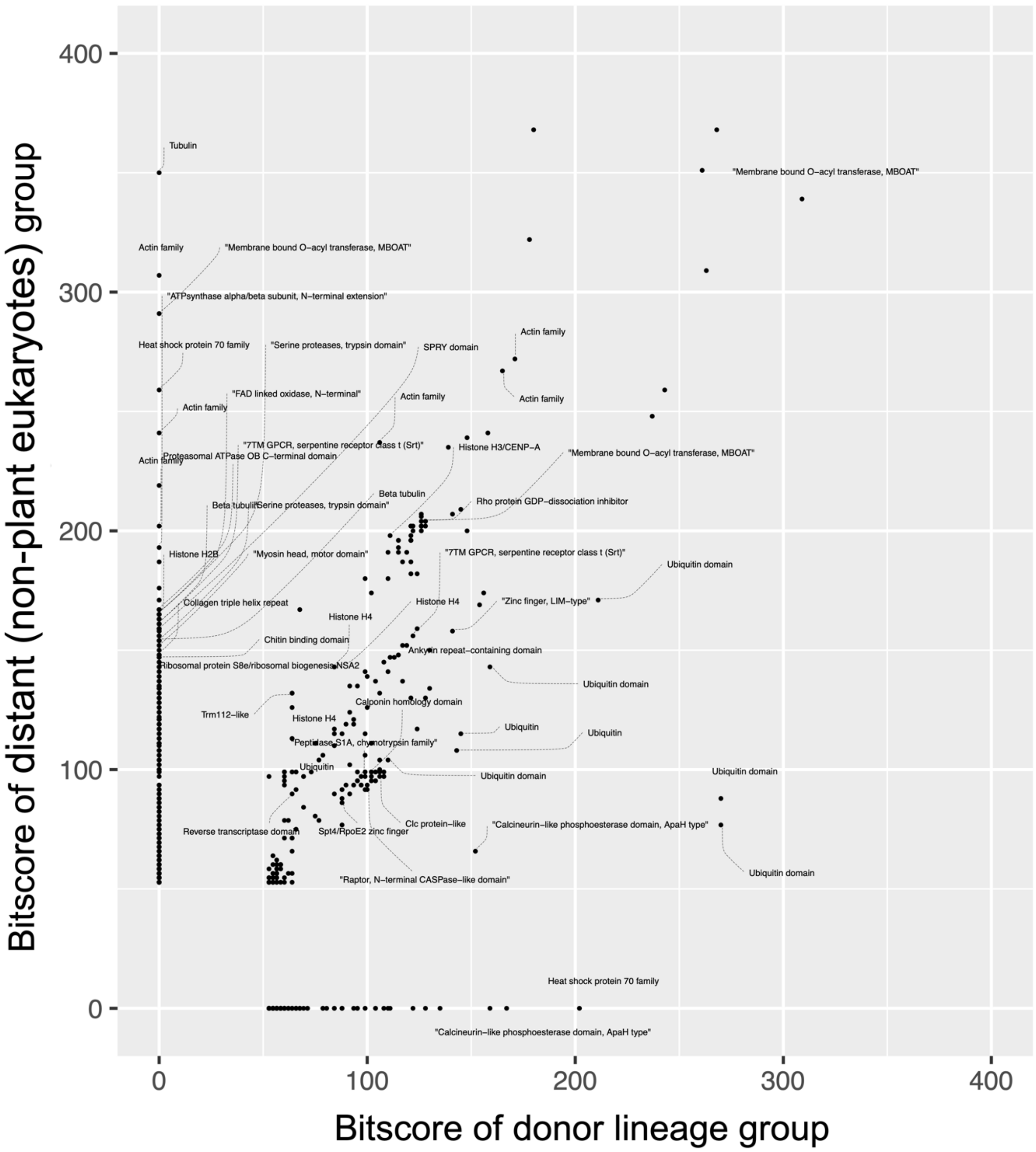

Figure S2. The similarity (bitsocre) comparison of 500bp-windows of the *H. schachtii* genome to putative donor lineages. X-axis: the bitscore from donor lineage group. Y-axis: the bitscore from distant group.

Gene_ID	AI	query	Best donor group hit bitscore	Best donor group hit identity	Best donor group hit alignment length	Best distant group hit bitscore	Best distant group hit identity	Best distant group hit alignment_length	Pfam/PRINTS ID	Pfam/PRINTS.description	Interpro.id	Interpro.description
Hsc_gene_20056	0.23	Hsc_scaff004_1760.0	270	87.288	236	76.8	91.071	56	PF00240	Ubiquitin family	IPR000626	Ubiquitin domain
Hsc_gene_20056	NA	Hsc_scaff004_1759.5	270	87.288	236	87.9	90.909	66	PF00240	Ubiquitin family	IPR000626	Ubiquitin domain
Hsc_gene_9882	0	Hsc_scaff001_1728.0	202	83.333	222	0	0	0	PR00301	70kDa heat shock protein signature	IPR013126	Heat shock protein 70 family
Hsc_gene_14856	-1.59	Hsc_scaff013_889.0	167	87.943	141	0	0	0	PF00149	Calcineurin-like phosphoesterase	IPR004843	"Calcineurin-like phosphoesterase domain, ApaH type"
NA	NA	Hsc_scaff017_104.5	159	87.143	140	0	0	0	NA	NA	NA	NA
Hsc_gene_14856	NA	Hsc_scaff013_888.5	152	86.765	136	65.8	93.182	44	PF00149	Calcineurin-like phosphoesterase	IPR004843	"Calcineurin-like phosphoesterase domain, ApaH type"
NA	NA	Hsc_scaff017_122.5	135	90	110	0	0	0	NA	NA	NA	NA
Hsc_gene_15956	-460.52	Hsc_scaff019_145.5	128	83.688	141	0	0	0	NA	NA	NA	NA
Hsc_gene_15956	NA	Hsc_scaff019_146.0	122	86.607	112	0	0	0	NA	NA	NA	NA
NA	NA	Hsc_scaff094_355.5	111	84.348	115	0	0	0	NA	NA	NA	NA
Hsc_gene_9882	NA	Hsc_scaff001_1729.0	104	85.294	102	0	0	0	PR00301	70kDa heat shock protein signature	IPR013126	Heat shock protein 70 family
Hsc_gene_7191	-13.99	Hsc_scaff014_489.0	95.3	78.571	154	0	0	0	PF03953	Tubulin C-terminal domain	IPR018316	"Tubulin/FtsZ, 2-layer sandwich domain"
NA	NA	Hsc_scaff014_485.0	95.3	78.571	154	0	0	0	NA	NA	NA	NA
Hsc_gene_4135	-0.72	Hsc_scaff061_627.5	87.9	80.702	114	0	0	0	PR00348	Ubiquitin signature	IPR019956	Ubiquitin
Hsc_gene_9882	NA	Hsc_scaff001_1729.5	78.7	79.032	124	0	0	0	PR00301	70kDa heat shock protein signature	IPR013126	Heat shock protein 70 family
Hsc_gene_12526	0	Hsc_scaff002_1937.5	67.6	76.86	121	0	0	0	PF00171	Aldehyde dehydrogenase family	IPR015590	Aldehyde dehydrogenase domain
Hsc_gene_25623	-21.55	Hsc_scaff006_2742.5	60.2	75.758	132	0	0	0	PF00149	Calcineurin-like phosphoesterase	IPR004843	"Calcineurin-like phosphoesterase domain, ApaH type"

Table S2. A table of 17 genomic windows showing higher similarity to a putative donor lineage group.

Contamination removal

Confident removal of contamination is of critical importance to HGT definition. Since the cyst nematode embeds its body inside the root tissue, nucleic acids from plants may be attached to the outside of nematode samples when collecting the nematodes from the host root. Besides carefully washing the nematode and using a plant-specific primer to test for contamination using PCR, a bleach solution was used to clean the surface of nematodes and to degrade attached plant DNA/RNA residuals. Sodium hypochlorite was known for digesting unwanted nucleic acids residues on the surface of fossil records (Kemp and Smith, 2005; Champlot et al. 2010). However, exposing nematodes to bleach solution for few minutes caused the nematode cuticular layers to peel off and the body contents to haemorrhage (Esser 1972), which affected the recovery rate of DNA extraction for the downstream experiment. To determine the most appropriate bleach concentration and soaking time that efficiently removes contaminating external nucleic acids without killing the nematode, female *H. schachtii* were immersed in a range of beach concentrations. When immersing the cysts into 0.1% to 1 % (w/v) of sodium hypochlorite solution for 5-10 minutes, the nematodes were not ruptured (Figure S3), and the surface DNA/RNA residual are undetectable (Figure S3 and Kemp and Smith, 2005).

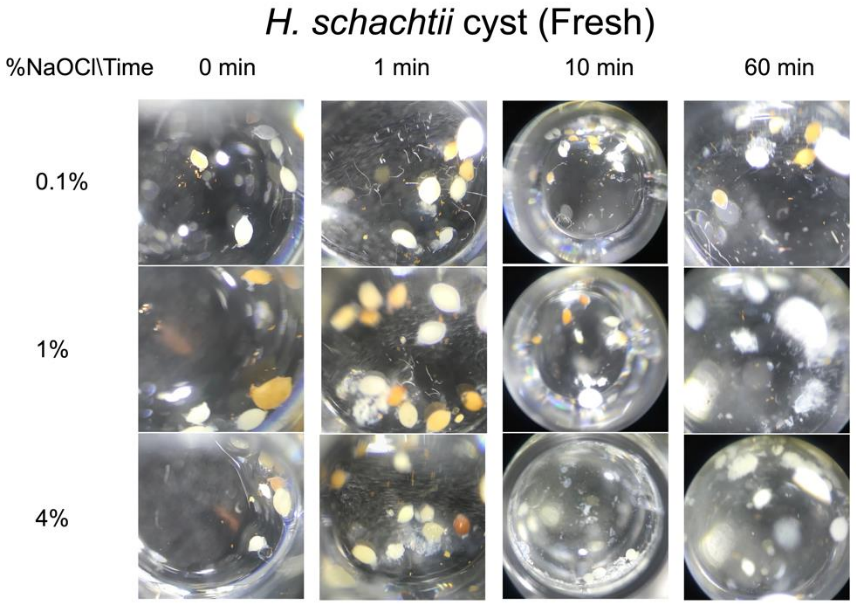

Figure S3. *H. schachtii* females soaked in three different bleach solutions. Each cyst is around 0.5mm - 0.8mm in length. In 0.1% (v/v) NaOCl solution, the first cyst burst after 1 minute, and approx. half burst after 10 minutes. In 1% (v/v) NaOCl solution, all cysts visibly shrunk within 10 mins. and contents hemorrhaged from their bodies. In 4% (v/v) NaOCl solution for a minute, after the cuticle degraded, cysts gradually turned white, surrounded by bubbles, and later dissolved.

To determine the efficacy of surface contamination removal a chloroplast gene the 5'trnL(UAA)-trnF(GAA) (abbreviated to trnL-F) was used (Koch et al. 2005). Due to their circular structure, the plastid genome will amplify by RCA, but it is deemed unlikely to transfer due to its size and sequestration in plastids. The primer pair was used to amplify the trnL-F region (Taberlet et al. 1991). The amplicons of trnL-F were identified in untreated nematode female samples but not in the bleached treated nematode samples (Figure S4). The SQT-1 like nematode gene was used to determine whether internal nematode DNA remained following external contamination removal (Figure S4).

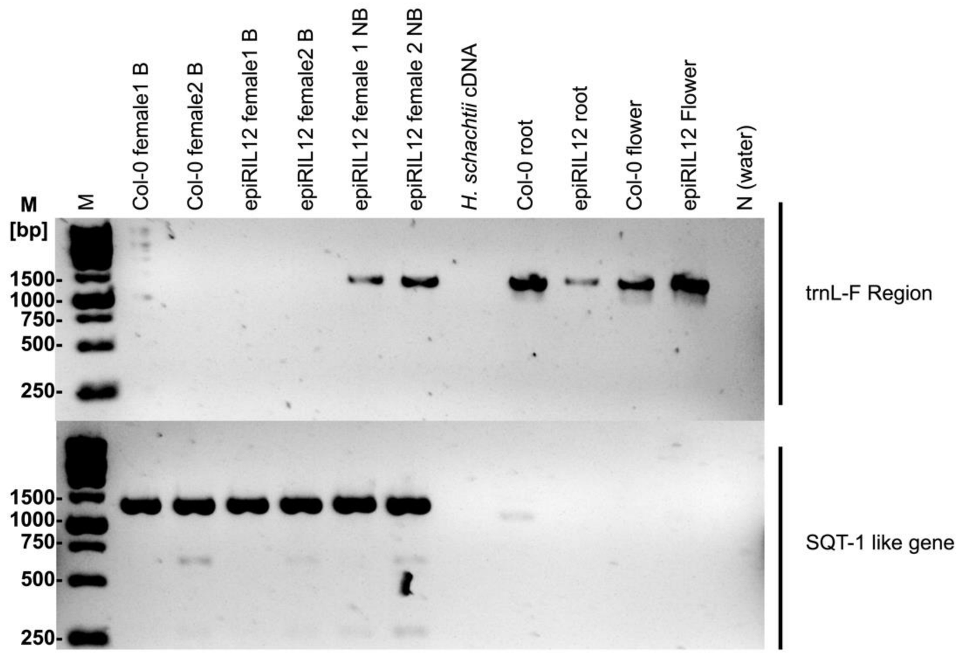

Figure S4. Gel electrophoresis of trnL-F plant-derived and SQT-1 like nematode-derived amplicons from bleached treated or untreated *H. schachtii* female cyst gDNA samples. “B” indicates bleached samples and “NB” indicates the non-bleached samples.

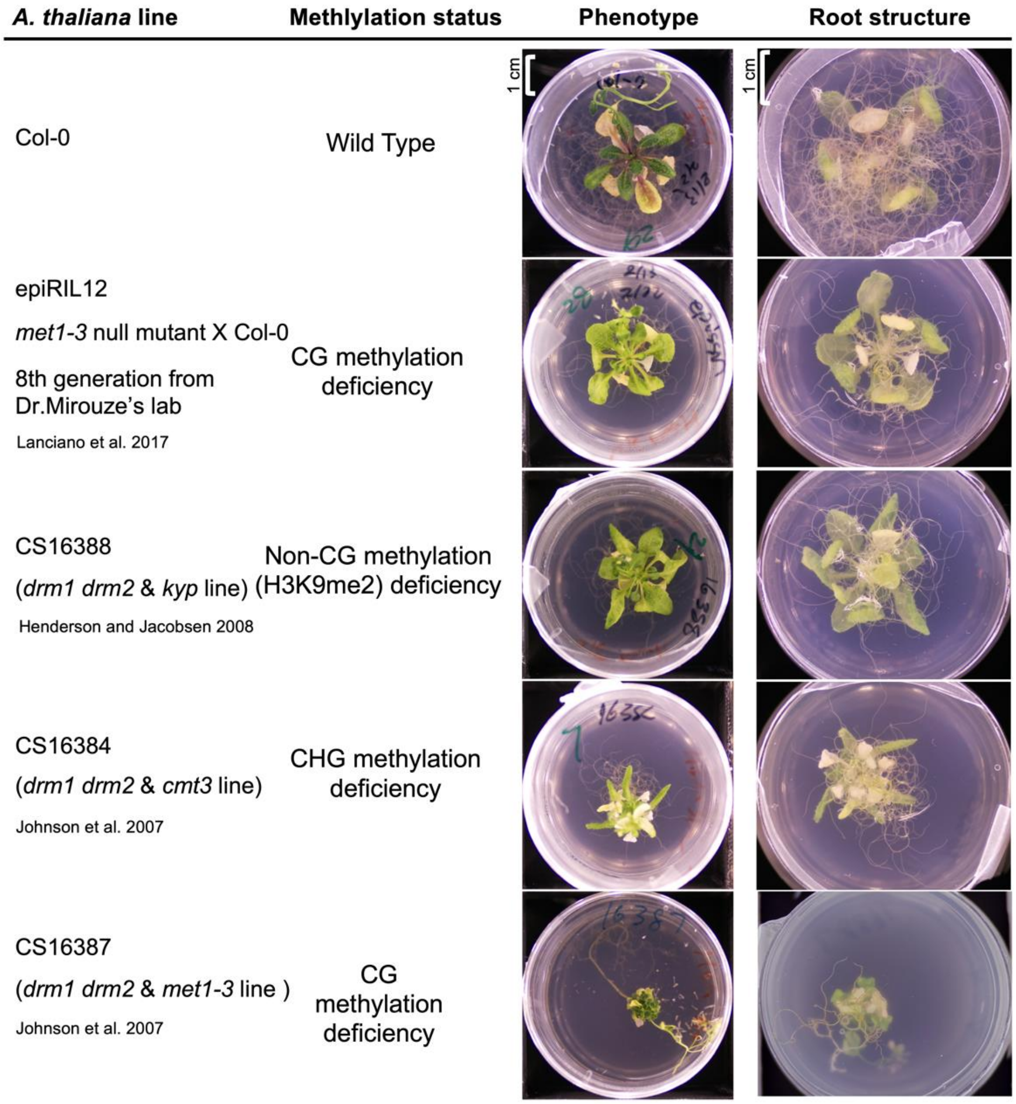

Figure S5. Each A. thaliana line 14 days post *H. schachtii* infection. The shoot and root phenotypes are visualized (pictures in the same Arabidopsis line came from different plates and are just examples). The scales represent 1 centimeter in length.

Demethylated regions in syncytium in Col-0 overlap with the eccDNA regions in epiRIL12

The overlap between the eccDNA produced in hypomethylated *A. thaliana* line, epiRIL12 (Lanciano et al. 2017), and the hypomethylated genomic regions in *H. schachtii* infected Col-0 *A. thaliana* (Hewezi et al. 2017) were inspected. Seven overlapped eccDNA/TEs were found in Table S3. This result indicated that *H. schachtii* is likely to induce the production of eccDNA in *A. thaliana*.

TE name	Locus	Super family	Transposon family
AT1TE41585	AT1G34780	DNA/MuDR	ATDNAI127T9C
AT1TE45800	AT1G36756	LTR/Gypsy	ATLANTYS1
AT3TE47280	AT3G29450	LTR/Copia	ATCOPIS41
AT4TE16165	AT4G06598	LTR/Gypsy	ATLANTYS1
AT4TE13555	AT4G05590	LTR/Gypsy	ATLANTYS1
AT4TE18315	AT4G07740	LINE/L1	ATLINE2
AT5TE20395	AT5G17120	LTR/Copia	ATCOPIS93

Table S3. Seven overlapped eccDNA/TEs found in both hypomethylated *A. thaliana* line, epiRIL12, and hypomethylated wild type *A. thaliana* (Col-0) in response to *H. schachtii* infection.

A

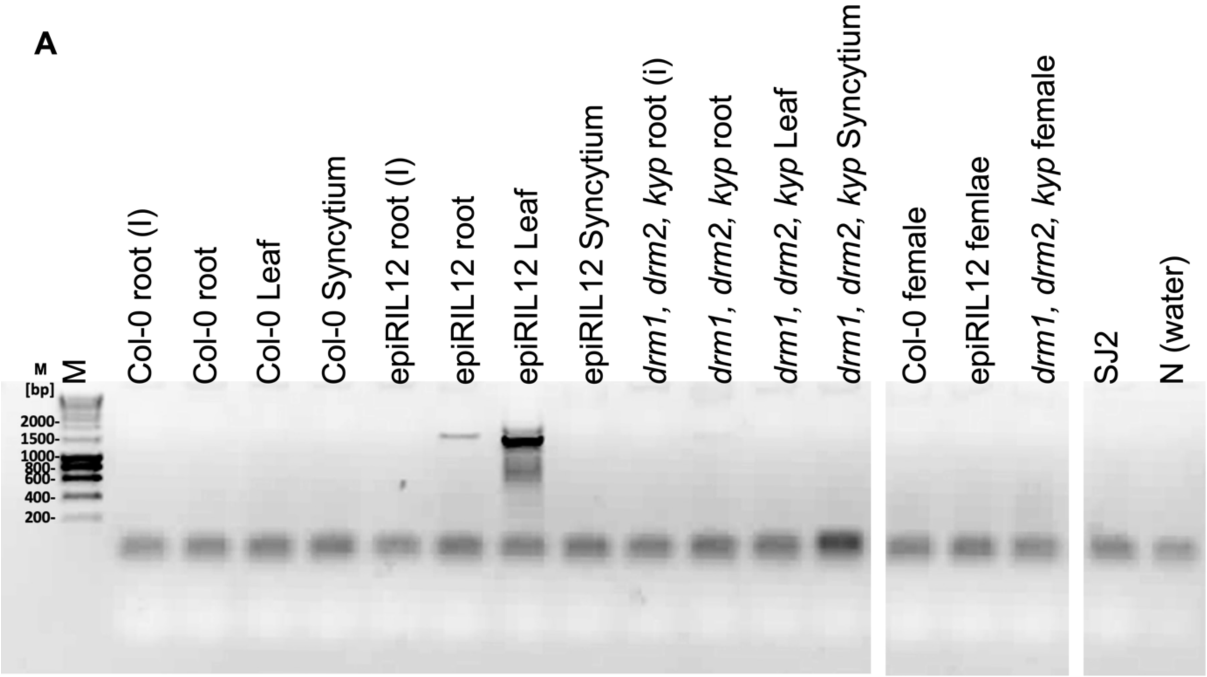

B

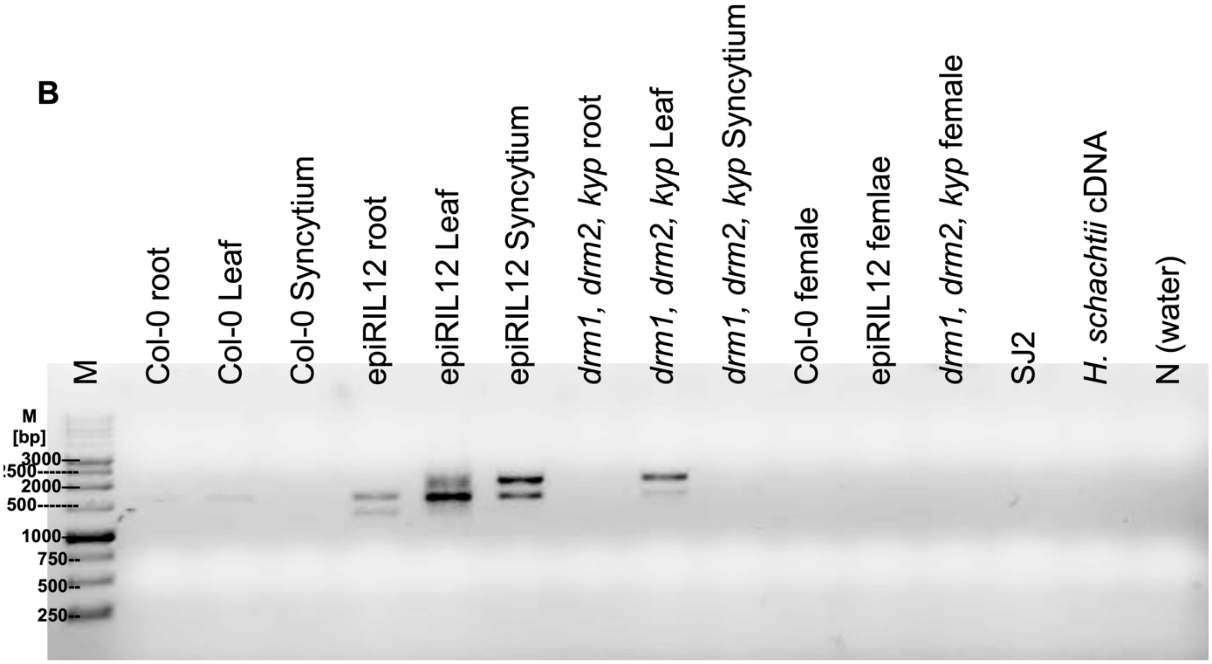

Figure S6. The amplicon of *EVADE/COPIA93* TE, “AT5TE20395” eccDNA from all samples. A) The above gel electrophoresis was performed before RCA. B) below gel electrophoresis was performed after RCA. “M” represents the maker. “I“ indicates the root tissues collect next to the syncytia.

**Library**	Col-0 *A. thaliana*				epiRIL12 *A. thaliana*				*H. schachtii*					
	Root		Syncytium		Root		Syncytium		WT female		epi12 female		J2	
**Analysis**	#1	#2	#1	#2	#1	#2	#1	#2	#1	#2	#1	#2	#1	#2
Library size (Pairs)	9.47M	9.49M	8.45M	8.78M	8.05M	10.41M	7.26M	9.28M	13.44M	6.80M	18.45M	7.18M	9.06M	6.79M
map to chloroplast	0.45M	0.17M	0.69M	1.02M	0.52M	0.34M	0.84M	0.82M	1030	12	4	3	58	15
map to mitochondria	4.44M	0.27M	2.42M	3.95M	0.92M	0.38M	1.64M	0.39M	3	14	0	1	2	36
Map to TAIR10 (*A. thaliana*)	1.55M	8.95M	3.41M	3.42M	5.59M	9.57M	4.36M	7.88M	1454	3970	3029	3371	1856	3025
%Arabidopsis mapped	67.99%	99.00%	77.22%	95.58%	87.19%	98.84%	94.19%	98.03%	0.02%	0.06%	0.02%	0.05%	0.02%	0.05%
Map to Cam v1.2 (*H. schachtii*)	1606	389	4009	7400	7843	1091	4909	58551	11.30M	5.96M	16.86M	6.31M	8.26M	5.97M
%*H. schachtii* mapped	0.02%	0.00%	0.05%	0.08%	0.10%	0.01%	0.07%	0.63%	84.09%	87.67%	91.40%	87.83%	91.19%	87.83%
unmapped reads	3.03M	0.09M	1.92M	0.38M	1.02M	0.12M	0.42M	0.12M	2.14M	0.83M	1.58M	0.87M	0.80M	0.82M
A. thaliana eccDNA identified Circle_Map (Prada-Luengo et al. 2019)	8271	26	12285	3906	34669	1023	22626	4688	0(2)	2	2(5)	2	0	0
Average base coverage within the circular DNA detection coordinates	47.6884	25.4821	22.4137	24.7499	56.9323	22.0088	40.0176	18.7958	0(7.62823)	36.2864	27.5875(19.8217)	43.8444	0	0
A.thaliana eccDNA identified ecc_finder (Zhang et al. 2021)	962	12	1142	271	3851	28	2668	37	1(1)	1	1(2)	1	0	1
Average supported discordant reads per eccDNA	62.9917	395.25	13.4947	40.3506	45.5944	127.464	36.0307	96.2432	38(47)	55	87(35.5)	96	0	1
Average supported split reads per eccDNA	89.238	36.25	28.0683	41.5498	103.547	24.8214	61.8819	40.4865	1(1)	1	1(1.5)	2	0	1
H. schachtii eccDNA identified Circle_Map (Prada-Luengo et al. 2019)	1	0	12	8	6	0	27	16	12093(11417)	11417	25115(25753)	19130	1492	3544
Average base coverage within the circular DNA detection coordinates	1.12351	0	32.3789	14.5036	1476.55	0	31.9109	17.1554	365.29(352.092)	49.5877	183.809(170.031)	91.4442	118.687	77.4507
H. schachtii eccDNA identified ecc_finder (Zhang et al. 2021)	0	0	5	6	3	0	21	12	1997(1909)	2297	3425(3425)	3367	421	605
Average supported discordant reads per eccDNA	0	0	3	6.16667	9.66667	0	11.8095	1.5833	103.257(347.409)	82.0296	71.4705(73.5442)	95.523	41.285	82.6281
Average supported split reads per eccDNA	0	0	8	7.5	9	0	21.619	2	358.439(347.409)	60.8659	241.669(248.914)	116.731	37.601	151.717

Table S4. Statistics of mobilome-seq libraries.

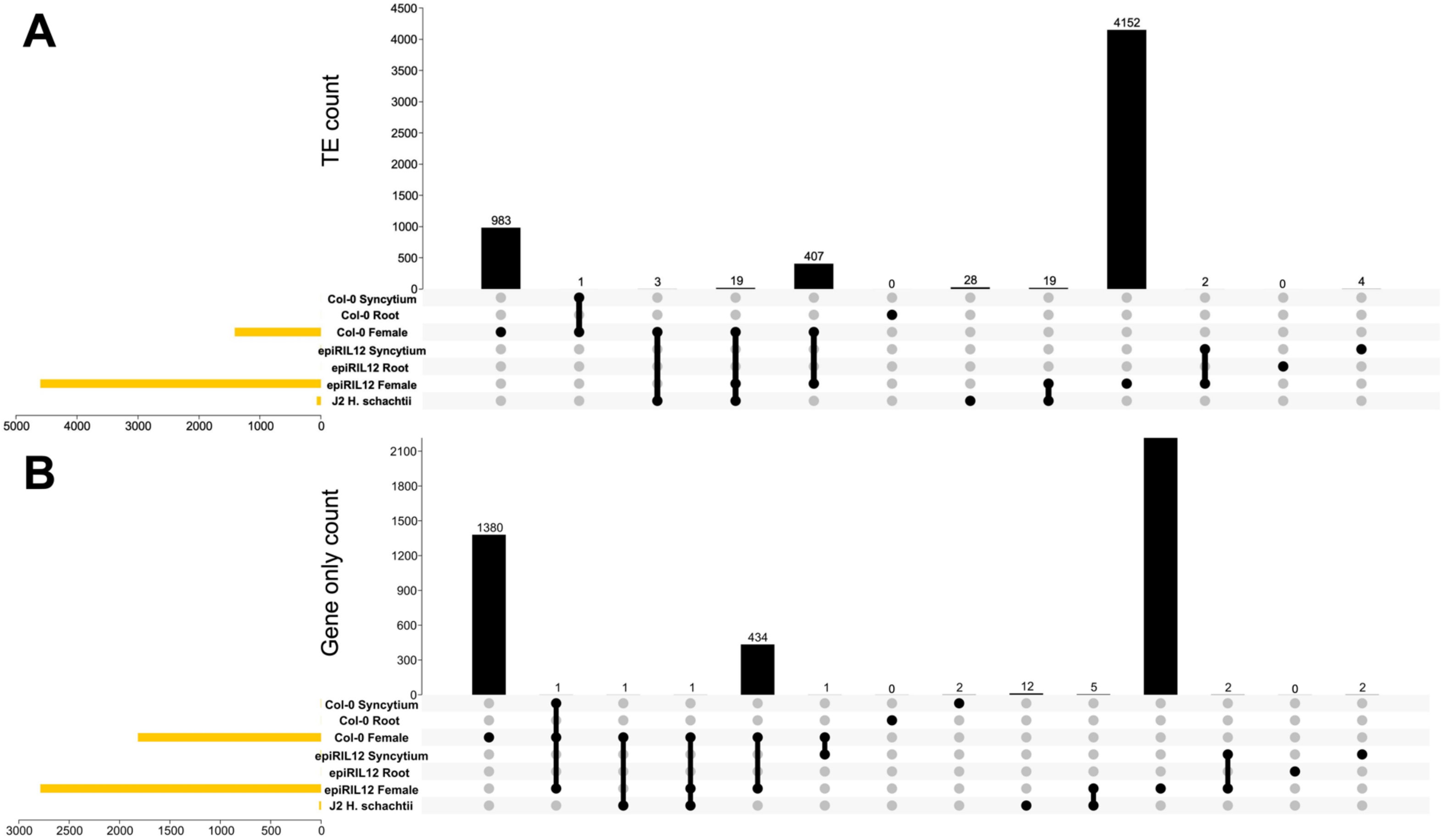

Figure S7. Nematode-derived eccDNA. Upset charts to show the overlap between *H. schachtii* TEs-derived **(A)** and gene-derived **(B)** eccDNA in different tissue types. Y-axis refers to TE counts and gene only counts. The number on the bottom left of each plot indicates the total eccDNA counts in each sample.

ASSAY	TARGET	FORWARD SEQUENCE (5'-3')	REVERSE SEQUENCE (5'-3')	AMPLICON SIZE (BP)	TM (°C)
Targeted approach to detect eccDNA movement from the plant to the nematode	AT5TE20395	TTGAAGTGTGTCGCTCTAATGCTGG	GCACAAACGGACTGATGAATAAAGC	1949 / 1543 bp	68
Untargeted approach to detect eccDNA movement from the plant to the nematode	AT2TE07175 (inbound)	AAGGACCAGCAGAATCACCT	CAGGATTTGGTCACGATGCA	2213bp	66
Untargeted approach to detect eccDNA movement from the plant to the nematode	AT2TE07175 (outbound)	AGGTGATTCTGCTGGTCCTT	TGCATCGTGACCAAATCCTG	2307bp	66
Contamination test: positive control for nematodes	sq1f/sq1r	CCTCGGCGTAATGAAAGTGT	CTCAGCCCGATCGCTGT	1295	66
Contamination test: positive control for plants	trn-LF_C/trn-LF_F	CGAAATCGGTAGACGCTACG	ATTTGAACTGGTGACACGAG	1353	63

Table S5. The table of primers used in this study. The primers were ordered from Sigma Aldrich, UK

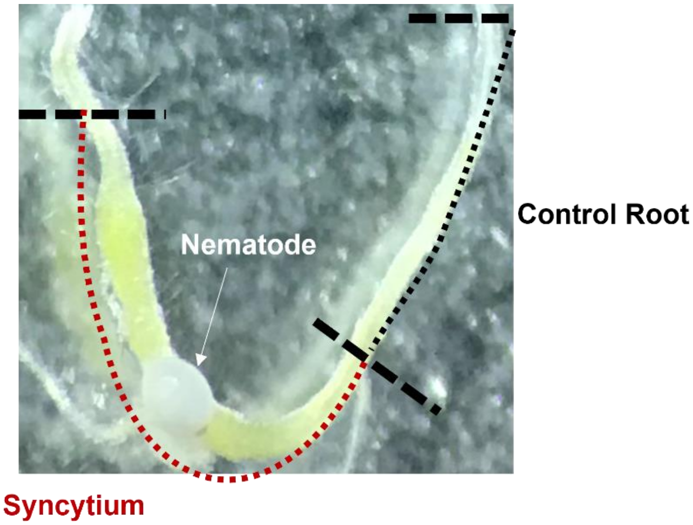

Figure S8. Representative image of sampling procedure for infected (Syncytium, red) and control root samples (black).

Supplemental references

Champlot S, Berthelot C, Pruvost M, Bennett EA, Grange T, Geigl E-M. 2010. An efficient multistrategy DNA decontamination procedure of PCR reagents for hypersensitive PCR applications. *PLoS One* [Internet] 5. Available from: http://dx.doi.org/10.1371/journal.pone.0013042

Esser RP. 1972. Effect of sodium hypochlorite concentrations on selected genera of nematodes. *Proc. Helminthol. Soc. Wash.* [Internet]. Available from: http://bionames.org/bionames-archive/issn/0018-0130/39/108.pdf

Henderson IR, Jacobsen SE. 2008. Tandem repeats upstream of the Arabidopsis endogene SDC recruit non-CG DNA methylation and initiate siRNA spreading. *Genes Dev.* 22:1597–1606.

Hewezi T, Lane T, Piya S, Rambani A, Rice JH, Staton M. 2017. Cyst Nematode Parasitism Induces Dynamic Changes in the Root Epigenome. *Plant Physiol.* 174:405–420.

Johnson LM, Bostick M, Zhang X, Kraft E, Henderson I, Callis J, Jacobsen SE. 2007. The SRA methyl-cytosine-binding domain links DNA and histone methylation. *Curr. Biol.* 17:379–384.

Kemp BM, Smith DG. 2005. Use of bleach to eliminate contaminating DNA from the surface of bones and teeth. *Forensic Sci. Int.* 154:53–61.

Koch MA, Dobes C, Matschinger M, Bleeker W, Vogel J, Kiefer M, Mitchell-Olds T. 2005. Evolution of the trnF(GAA) gene in Arabidopsis relatives and the brassicaceae family: monophyletic origin and subsequent diversification of a plastidic pseudogene. *Mol. Biol. Evol.* 22:1032–1043.

Lanciano S, Carpentier M-C, Llauro C, Jobet E, Robakowska-Hyzorek D, Lasserre E, Ghesquière A, Panaud O, Mirouze M. 2017. Sequencing the extrachromosomal circular mobilome reveals retrotransposon activity in plants. *PLoS Genet.* 13:e1006630.

Prada-Luengo I, Krogh A, Maretty L, Regenberg B. 2019. Sensitive detection of circular DNAs at single-nucleotide resolution using guided realignment of partially aligned reads. *BMC Bioinformatics* 20:663.

Taberlet P, Gielly L, Pautou G, Bouvet J. 1991. Universal primers for amplification of three non-coding regions of chloroplast DNA. *Plant Mol. Biol.* 17:1105–1109.

Yang Z, Zhang Y, Wafula EK, Honaas LA, Ralph PE, Jones S, Clarke CR, Liu S, Su C, Zhang H, et al. 2016. Horizontal gene transfer is more frequent with increased heterotrophy and contributes to parasite adaptation. *Proc. Natl. Acad. Sci. U. S. A.* 113:E7010–E7019.

Zhang P, Peng H, Llauro C, Bucher E, Mirouze M. 2021. ecc_finder: A Robust and Accurate Tool for Detecting Extrachromosomal Circular DNA From Sequencing Data. *Front. Plant Sci.* 12:743742.
